# The optimal route search in Bengaluru city transport using Hamilton circuit algorithm

**DOI:** 10.1016/j.heliyon.2025.e42519

**Published:** 2025-02-07

**Authors:** Parkavi S, Parthiban A

**Affiliations:** Department of Mathematics, School of Advanced Sciences, Vellore Institute of Technology, Vellore, 632 014, Tamil Nadu, India

**Keywords:** Graph theory, Hamiltonian circuit algorithm, Transportation, Optimal route, Backtracking approach

## Abstract

Traffic congestion is a common transportation issue all over the globe, especially in India's large cities. One of the primary causes of traffic congestion is the sheer volume of private vehicles that travel on city streets and the extensive use of public transportation. The lack of additional road capacity also makes it worse. As a result, many urban road traffic situations, particularly in Bengaluru city in India, are approaching saturation, characterized as the situation where the number of cars using a road is equal to its capacity (V/C=1). One strategy to address the issue of congestion is traffic engineering. So, drawing inspiration and motivation from the outstanding work of Mungporn, Pongsiri et al., “Modeling and control of multiphase interleaved fuel-cell boost converter based on Hamiltonian control theory for transportation applications, IEEE Transactions on Transportation Electrification 6.2, 2020, pp. 519-529”, in this paper, we study an intelligent agent model to perform route engineering for public transportation in the Bengaluru city, based on the Hamilton circuit algorithm and analyze the best and optimal route among the three significant routes out of twelve available using various parameters. The public transit route is changed from being a single path connecting the outgoing and returning routes to a circular route. The results of the simulation indicate that the reconfigured circular routes, optimized using the backtracking algorithm, led to a notable reduction in the traffic load on public transportation. This optimization improves overall traffic flow and alleviates congestion in key areas of Bengaluru.

## 1 Introduction

The quantity of automobiles keeps increasing every day in the major cities in India, especially in metro cities. One strategy for reducing the density of highways is the use of traffic engineering. The focus of the study undertaken is public transit in urban areas, and the debate is centered on highway traffic in Bengaluru. The abundance of city transit options in Bengaluru is widely recognized. This undoubtedly plays a part in the city's traffic jams. From an information technology standpoint, it is possible to use a computer to perform traffic engineering simulations on an urban transportation route to effectively design city transit paths that minimize traffic.

In the realm of information technology, computer-based simulations can be employed for traffic engineering on urban transport routes. This approach facilitates the optimization of city transport route planning to alleviate congestion and enhance overall efficiency. A majority of research studies conducted in the realm of highway traffic modeling have employed graph concepts as the fundamental basis [1]. They have extensively advocated a graph-based model design to control the flow of traffic. Graph theory is applied to represent and enhance travel routes within urban public transport systems. For instance, it has been utilized for traffic modeling in Indian cities [11] and the analysis of bus routes in Brazilian urban areas [8]. Graphs are utilized for representing air traffic as well, as demonstrated in [9].

Based on information sourced from the Karnataka Road Transport Corporation, Bengaluru had a greater quantity of both motorized two- and four-wheeled vehicles in 2020 compared to previous records. The city's transportation department has observed an increase in the overall count of motorized vehicles. JP Nagar is a neighborhood located within the Bengaluru urban district of Karnataka state, boasting a population of 282,664 individuals. Among them, there are 150,449 males and 132,215 females. The area covers approximately 13.94 square kilometers. The Hebbal Flyover poses a significant traffic obstacle in the northern section of Bengaluru. With its narrow lanes and heavy traffic volume, congestion is a common occurrence, resulting in lengthy delays and frequent accidents. One of the busiest areas in the city lies between JP Nagar and Hebbal, a renowned route where we carefully select the optimal transportation route to navigate through traffic. This involves analyzing both peak and off-peak hours during weekdays and weekends, effectively dividing the day into two distinct periods for travel planning.

The number of vehicles (V) and the capacity (C) of the road to handle traffic are the two primary criteria that affect the standards for traffic density on a certain stretch of the route. If the V/C ratio falls within the range of 1.0 to 1.2, after which the road segment's level of traffic is considered typical. However, when the ratio V/C equals 1, it indicates that the road is considered saturated, resulting in congestion due to heavy traffic flow. When the V/C ratio is more than 1, overload situations arise. In the coming years, there will be greater challenges ahead as a result of the anticipated rise in the number of automobiles. Nonetheless, there are still opportunities to address future congestion effectively.

To create a model of the highway traffic system, graphs are used to represent the connections between various points with extreme precision. In this study, the conceptual framework for alternate urban transit routes in Bengaluru was developed using the Hamilton circuit method. Through this method, it might be possible to optimize the one-lane transit paths in Bengaluru into a circular route, allowing for an evenly distributed number of urban transport lanes rather than a single lane. This can, of course, lessen the saturation level (V/C) of the route that municipal transit frequently uses.

In transportation engineering, [6] the “V/C ratio” signifies the relationship between traffic volume (V) and capacity (C) of a road or transportation infrastructure. It is a crucial measure for evaluating congestion or saturation levels within a transportation network. Here is the equation:

(1) $$V/C=\frac{\text{Volume of traffic}}{\text{Capacity of the transportation facility}}$$

In the above Equation (1), V indicates the real amount of vehicles or passengers utilizing the transportation infrastructure during a designated period, such as the number of vehicles per hour or passengers per hour; C refers to the highest capacity of traffic that the transportation facility can effectively manage without issues during regular circumstances within a given period.

The utilization of transportation infrastructure [6] can be inferred from the V/C ratio. When the ratio is greater than 1, it indicates that there is more traffic than there is capacity, which may indicate congestion and potential delays. On the other hand, if the ratio is less than 1, it implies that the infrastructure is not being completely exploited and that the volume of traffic is below capacity. Assess the efficiency of the current transportation system, identify regions that are crowded, forecast future demand, and make necessary plans to adapt to expected rises in traffic while maintaining acceptable service levels.

## 2 Related works

To analyze and optimize transportation networks, [12] presented graph theory parameters and algorithms. These included the use of the SPEA algorithm to solve the 0-1 knapsack problem, minimal spanning tree techniques, and accurate domination parameter problems. A multi-phase interleaved boost converter with a Hamiltonian function-based control strategy was created by [2] specifically for dynamic transportation applications. Their study uses digital computations and simulations to verify the efficacy of the suggested control algorithm in managing extremely dynamic power-load cycles. It contains a 2.5 KW two-phase interleaved converter coupled with a methanol fuel cell system. By solving the Hamiltonian cycle issue for the whole graph using the quantum circuit of the Grover algorithm, [3] created explicit and implicit oracles for quantum algorithms and showed a quadratic acceleration over classic unorganized search strategies. Symbolic matrices and an algebraic approach were introduced by [4] to identify Hamiltonian circuits in a basic unoriented graph and to list spanning trees. Examples of a concrete, fully parallel algorithm that accomplishes both objectives are provided. Additionally, a sufficient and necessary condition for Hamiltonicity is given. The goal of the Internet of Things project is to integrate control and sensing into networks. Bond graphs can be used to simulate several processes in various domains, as demonstrated [5] Input-State-Output Port-Hamiltonian (ISO PHS) formulations are used in this work to build mathematical models for non-linear sensing and actuation systems. The translation of bond graph representations that take into consideration energy exchange between various network ports is the main topic, with applications in sensor network architecture. Graph theoretic models, as stated [10] may be an effective way to address intersection traffic control issues. By reducing waiting times and sensor costs, they propose that compatibility graphs connectedness can effectively direct traffic flow, making this strategy applicable to a variety of junctions. A novel method for employing artificial intelligence (AI) to find fuzzy Hamiltonian cycles in fuzzy Hamiltonian networks was presented [28] when adjacency matrices describe graphs, it is easy to determine whether an edge connects two vertices. Furthermore, the study displays techniques that make use of the chosen fuzzy graph trip system with AI structure.

In [17] developed a solution for a practical problem by applying Kruskal's algorithm to find a minimum spanning tree and using Dijkstra's algorithm to determine the shortest path between two points. Additionally, they created a network model for the transportation problem, which was thoroughly analyzed to reduce shipping costs. As suggested by [18] an Ant-like Method based on graphs for optimal path planning. An ant colony optimization (ACO) technique in conjunction with a graph-based search algorithm was used to create a novel hybrid model that optimized the trajectory of a mobile robot's global travel. Using MAKLINK graph theory to describe the robot workspace and obstacles, the Dijkstra algorithm is then used to create a suboptimal itinerary for the robot that avoids collisions. ACO and a smoothing technique based on B-spline curves are used to optimize the global sub-optimal trajectory generated by the Dijkstra algorithm in Cartesian space. The effectiveness of the proposed hybrid graph-based model is demonstrated through simulation and comparative studies in diverse environments. An extended Dijkstra algorithm for surface optimal path planning was introduced by [19]. By creating a two-dimensional triangle from the triangle mesh on the surface, the Delaunay triangulation is utilized to simulate the outer environment. By constructing a passable, [13] two-dimensional channel on the surface and solving for the best path inside it, the transformation finds the shortest path out of all the available optimal paths. This approach translates plane coordinates to surface coordinates, improving the surface optimization path accuracy in single-robot single-target and multi-robot multi-target path planning tasks. Scalable calculation of dynamic flow problems using multi-marginal graph-structured optimal transport was introduced [20].

By using optimal transport theory to describe the multi-commodity minimum-cost network flow problem as a multi-marginal optimal transport problem, the paper introduces an innovative framework for dynamic network flow problems. The effective projections made possible by the graph structure in Sinkhorn iterations result in a computationally efficient and simple-to-use algorithm that outperforms a Linear programming solver and works exceptionally well for traffic routing issues. A convex version of the classical issue was developed [21] in which the edge length is a convex function and each vertex's position is a continuous choice variable. A lightweight mixed-integer convex formulation with perspective operators is our primary contribution, which makes it possible to efficiently find globally optimal pathways in high-dimensional spaces and intricate, large-scale graphs. In [26] introduced a new framework for detecting attacks in RF-based Electronic Warfare (EW), identifying signal blockages for anti-jammer installations, and recommending optimal mission paths. It employs a logistic regression-based ML mechanism, a SARSA-based reinforcement learning scheme, and evaluates using a real-world EW dataset. The logistic regression approach demonstrates the potential of machine learning and graph theory in network security and optimization with its 98% accuracy in attack detection and path design. Reinterpreting transition state and path theories via the lens of optimum control, [27] established a data-driven approach for rare events in biological reactions. They computed a committer function and constructed a discrete Markov process, which resulted in an optimally regulated random walk on point clouds. An algorithm for efficient computation of the mean transition path, based on an optimally controlled random walk, has been developed. Numerical examples demonstrate that this data-driven solver aligns with the dominant transition path in transition path theory. Table 1 presents an analysis and comparison of models relevant to this study.

**Table 1:** Summary of the related works.

S.No	Reference	Contribution	Methodology	Limitation/Future work
1	[17]	Determines the best routes for the transportation problem to establish an MST	Kruskal's algorithm and Dijkstra's shortest path algorithm	Not widely employed in major algorithms and examined with a restricted range of techniques

2	[18]	The novel hybrid model has been developed to optimize the global path of mobile robots by employing a graph-based search algorithm and ACO method	Obstacles are modeled using MAKLINK graph theory and the Dijkstra algorithm	Utilizes the multi-optimal hybrid for implementation and the optimum path models for evaluation

3	[19]	Extended Dijkstra Algorithm optimizes surface paths by converting the triangle mesh into a two-dimensional triangle	Dijkstra algorithm	Traditional Dijkstra algorithm's use of Euclidean distances in surface path planning can lead to significant errors

4	[20]	A new framework uses optimal transport theory to address the multi-commodity minimum-cost network flow problem	Entropy-regularized optimal transport problems	In the new hybrid model, one can employ widely-used algorithms

5	[21]	Examines a variation in which edge lengths are convex functions of vertex positions, which are continual decision variables inside a convex set	Autonomous cars to NP-hard optimum control of hybrid systems such as SPP in GCS	Utilized a hybrid approach combining low-dimensional optimal path techniques with high-dimensional application

6	[26]	RF network assault detection and optimal route determination and investigate a new framework for proactively spotting signal obstructions, anticipating RF-based EW attacks, and suggesting the best mission pathways	RF-based Electronic Warfare (EW), state-action-reward-state-action (SARSA) reinforcement learning (RL)	Logistic regression achieves 98% accuracy in attack detection with a normalized Euclidean distance error of 0.1 to 0.3.

7	[27]	Recast transition state and path theories as infinite-horizon optimal control problems	A Markov process with Voronoi tessellation, consistent with the dominant transition path algorithm	Proving this consistency rigorously in mathematical terms is an interesting future problem

## 3 Methodology

**Figure 1:**
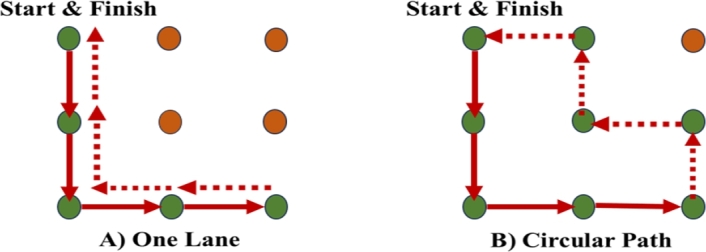
A single-lane route (A) and dissimilarity between a circular track route (B).

The study uses a mixture of the Hamilton circuit algorithm and intelligent agent models (intelligent agents) to optimize the flow of urban transit routes in Bengaluru. The purpose of using this strategy is to identify the best transit route, which may be improved over the existing one-lane route, by creating a circular path and providing the current route's greatest option (shown in Fig. 1-(A,B)).

### 3.1 Data

**Figure 2:**
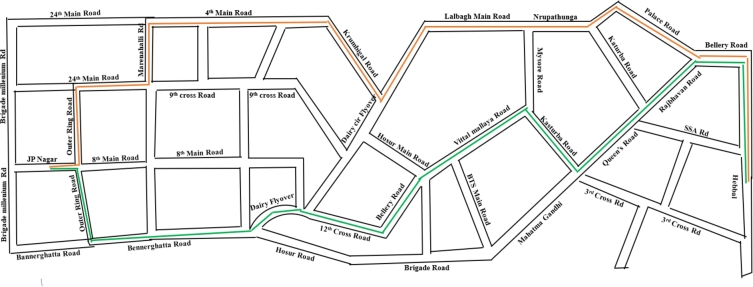
The pathway utilized by city transportation from “JP Nagar to Hebbal”.

The investigation makes use of data from the Bengaluru city transportation agency, which contains information on the names of the streets, city transportation routes, mileage, duration, gasoline, tolls, and saturation level (V/C). Using city transport, the investigators employed the JP Nagar-Hebbal urban transport route for simulation. As shown in Fig. 2, the route includes some urban transportation and travels 19.3 km to reach Hebbal from the starting point at JP Nagar, the terminal. Accordingly, the journey's back-and-forth mileage is 38.6 km. Except for a few change routes owing to one-sided routes, the way back from Hebbal travels via the identical path as the departure to JP Nagar, the terminal.

There are fifteen road segments to cross on the way from JP Nagar terminal to Hebbal and thirteen on the way back. Since the road is in a single direction, there are multiple routes to take when leaving and returning.

**Figure 3:**
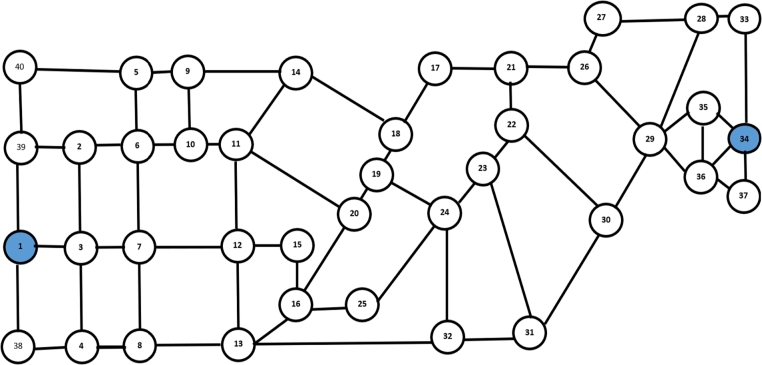
A graphic structure depicting Bengaluru city's road plan as a basis where JP Nagar terminal is the departure point at node No. 1, and Hebbal is the goal at node No. 34.

The road portions in Fig. 2 need to have been transformed into a graph to run the simulation, this can be done by first turning each intersection into a node. The next step is connecting each newly generated node to another node by assigning a line or vertex to that node. The conversion's outcomes are displayed in Fig. 3. Additionally, the weight of every point connecting two nodes is determined by the saturation ratio (V/C=1). The mass is a guiding criterion for creating different Hamilton circuit-based paths.

### 3.2 Hamiltonian circuit

A Hamiltonian circuit is a route that traverses every graph node exactly once. The term “Hamilton circuit” refers to a closed route that forms when a path returns to the initial point. A Hamilton circuit passes through each node in the graph specifically once, except the first and last nodes, which are traversed over twice.

Numerous alternate circuit paths can be chosen and followed, thanks to the Hamilton circuit. The number of Hamilton circuits that may be estimated via Equation (2), where *n* is the number of locations on the graph, and journeys to cities are determined by a certain criterion.

(2) $$N=\frac{1}{2}(n-1)!$$

**Figure 4:**
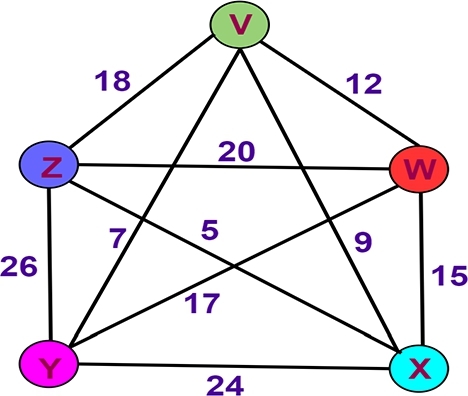
Graph with 5 nodes and 10 edges.

**Figure 5:**
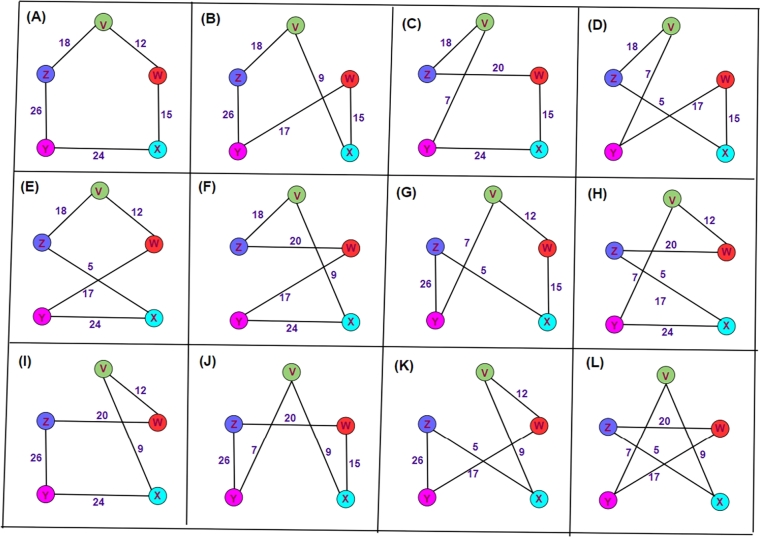
Twelve other closed paths, namely (A) *V* − *W* − *X* − *Y* − *Z* − *V*, (B) *V* − *X* − *W* − *Y* − *Z* − *V*, (C) *V* − *Y* − *X* − *W* − *Z* − *V*, (D) *V* − *Y* − *W* − *X* − *Z* − *V*, (E) *V* − *W* − *Y* − *X* − *Z* − *V*, (F) *V* − *X* − *Y* − *W* − *Z* − *V*, (G) *V* − *W* − *X* − *Z* − *Y* − *V*, (H) *V* − *W* − *Z* − *X* − *Y* − *V*, (I) *V* − *W* − *Z* − *Y* − *X* − *V*, (J) *V* − *X* − *W* − *Z* − *Y* − *V*, (K) *V* − *W* − *Y* − *Z* − *X* − *V*, and (L) *V* − *X* − *Z* − *W* − *Y* − *V* satisfy the requirements to be considered Hamilton circuits.

To illustrate Fig. 4, we are aware of 5 nodes (v,w,x,y, and *z*) and 10 weighted edges (18, 12, 15, 24, 26, 7, 20, 9, and 17). For instance, the ten lines indicate the ten roads that are to be traveled, and the four points indicate the five city boundaries that are to be investigated (the goal). Twelve alternative paths (collected from $\frac{1}{2}⁎(5-1)!=12$) that satisfy the requirements of the Hamilton circuit are going to be found if Equation (2) is used for the computation, as shown in Table 2. Fig. 5-((A)-(L)) illustrates the twelve different routes that can potentially be displayed.

**Table 2:** Twelve different routes that satisfy the requirements to be considered Hamilton circuits.

S.No	Hamiltonian circuit line	Total mass
1	(*v*,*w*,*x*,*y*,*z*,*v*) or (*v*,*z*,*y*,*x*,*w*,*v*)	94
2	(*v*,*x*,*w*,*y*,*z*,*v*) or (*v*,*z*,*y*,*w*,*x*,*v*)	76
3	(*v*,*y*,*x*,*w*,*z*,*v*) or (*v*,*z*,*w*,*x*,*y*,*v*)	88
4	(*v*,*z*,*x*,*w*,*y*,*v*) or (*v*,*y*,*w*,*x*,*z*,*v*)	84
5	(*v*,*z*,*x*,*y*,*w*,*v*) or (*v*,*w*,*y*,*x*,*z*,*v*)	58
6	(*v*,*z*,*w*,*y*,*x*,*v*) or (*v*,*x*,*y*,*w*,*z*,*v*)	68
7	(*v*,*w*,*x*,*z*,*y*,*v*) or (*v*,*y*,*z*,*x*,*w*,*v*)	62
8	(*v*,*w*,*z*,*x*,*y*,*v*) or (*v*,*y*,*x*,*z*,*w*,*v*)	85
9	(*v*,*w*,*z*,*y*,*x*,*v*) or (*v*,*x*,*y*,*z*,*w*,*v*)	91
10	(*v*,*x*,*w*,*z*,*y*,*v*) or (*v*,*y*,*z*,*w*,*x*,*v*)	65
11	(*v*,*w*,*y*,*z*,*x*,*v*) or (*v*,*x*,*z*,*y*,*w*,*v*)	77
12	(*v*,*x*,*z*,*w*,*y*,*v*) or (*v*,*y*,*w*,*z*,*x*,*v*)	69

The algorithm is used to optimize public transportation routes in Bengaluru city, improving efficiency and reducing congestion and helps to identify the optimal circular routes, aiding traffic management in high-density urban areas. The intelligent agent model provides advanced simulations, guiding city planners in designing effective public transit routes. This study contributes to traffic management strategies.

### 3.3 Backtracking approach

One common method for addressing challenges is the backtracking methodology. This method's search strategy is its foundation. In an attempt to obtain a potential set of responses, this method of searching is applied. An ideal or fulfilling answer will be found from this range of potential results. Many strategies for going backtrack have been employed, such as the Sum of subsets, Graph coloring, Hamilton cycles, Knapsack, Traveling salesman, and the Eight queens problems. The backtracking procedure is shown broadly in the Algorithm 1.

**Algorithm 1:**
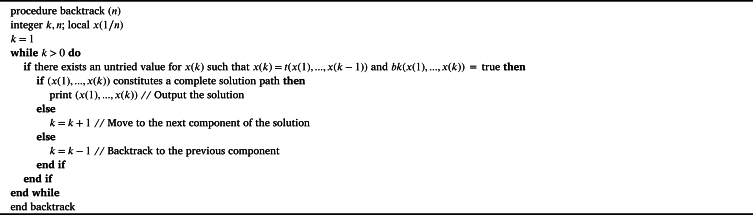
Backtracking Algorithm.

The backtracking approach helps to identify the best Hamilton circuit from the several options in the present investigation. Because this method of searching is continuous, finding and displaying the ideal Hamilton path requires some time. The route with the lowest overall weight in the V/C=1 ratio is called the ideal path.

The Hamiltonian circuit problem requires the use of backtracking strategies. The following justifies why going back is crucial for resolving the Hamiltonian circuit problem. Applying this algorithm to optimize routes in Bengaluru's transportation system, particularly for identifying Hamiltonian circuits, offers several advantages. It guarantees a comprehensive exploration of potential routes, addresses complex urban transportation challenges, and enhances efficiency through pruning and early termination techniques. It discards unfeasible routes and refines route configurations through incremental testing, minimizing congestion and maximizing road capacity use. This approach improves service quality and practical route planning by evaluating every possible path and selecting the most effective one. By employing mathematical methods, this algorithm effectively tackles real-world Bengaluru transportation issues, boosting operational efficiency and designing optimal transit routes for metropolitan areas.

#### 3.3.1 Exhaustive search

The Hamiltonian circuit problem entails an in-depth search for a loop that makes exactly one visit to each point. When an algorithm comes across dead ends or incorrect setups, it can retrace, which makes it a useful tool for methodically exploring every path that could exist.

#### 3.3.2 Permutation generation

To determine whether a Hamiltonian circuit exists, backtracking is utilized to create permutations of vertices. Backtracking assists in determining a valid Hamiltonian circuit by methodically permuting the vertices and determining whether an edge exists among successive vertices.

#### 3.3.3 Decision space exploration

In essence, solving the Hamiltonian circuit problem involves choosing a sequence of actions that result in a working circuit. Backtracking is a decision-making process that involves making decisions, going back as needed, and keeping going until a viable Hamiltonian circuit is discovered or all options have been considered.

#### 3.3.4 Avoiding redundant paths

If it becomes clear that the present path cannot be extended to a genuine Hamiltonian circuit, backtracking helps to eliminate repetitive paths by reducing the size of the region of search. The algorithm's temporal difficulty is greatly decreased by this trimming.

#### 3.3.5 Pruning method

In [22], an automatic vision transformer pruning method was introduced to simplify the deployment of models on resource-limited devices. Geographic routing systems can eliminate unnecessary hops by implementing an effective path pruning (PP) approach, as demonstrated in [23]. By employing wireless node channel listening to identify shortcuts, delivery rates were increased and routes were efficiently reduced. To determine the path with the shortest expected transit time in stochastic and time-dependent networks, a pruning-based technique was presented in [24]. Using a bi-level pruning criterion, the algorithm performs better on medium- and large-sized networks than previous approaches, and it excels on huge networks. To handle complexity and frequent data misclassification problems, a novel pre-pruning technique for decision trees was presented in [25].

Pruning enhances public transportation route planning by removing routes that don't meet performance standards, thereby reducing computational overhead improving efficiency by concentrating on viable options, and boosting the efficiency of both the Hamilton circuit algorithm and backtracking, which ultimately enhances the accuracy of route selection. This technique systematically eliminates less relevant choices, simplifying decision-making and focusing resources on the most important elements. In this paper, optimizing Bengaluru's public transportation routes is done in 4 stages as follows:

Initial route pruning: Filter out less promising routes using initial criteria such as traffic data and road capacity before implementing the Hamilton circuit algorithm.

Hamilton circuit pruning: When finding the optimal circular route with the Hamilton circuit algorithm, exclude paths that don't meet efficiency or traffic flow standards, and focus on viable options.

Backtracking algorithm pruning: Use pruning to eliminate routes that don't meet essential constraints during backtracking, thereby narrowing down to the most promising options.

Simulation pruning: After simulations, routes with minimal traffic flow improvement are discarded, and the top-performing routes, such as Route 1, are optimized.

#### 3.3.6 Optimization

By using strategies or methods to give some paths priority over others, backtracking can be minimized. Pruning methods can be used to find more effective solutions by removing parts of the search space that are unlikely to produce a Hamiltonian circuit.

In essence, backtracking is essential for solving the Hamiltonian circuit problem because it enables systematic exploration of potential solutions, adeptly manages constraints and enhances the efficiency of the search process. This method offers a potent strategy for discovering Hamiltonian circuits within graph structures.

## 4 Measuring traffic congestion

Congestion in urban traffic poses a significant challenge for transportation systems in cities. Different academics have offered varied views of traffic congestion. However, there isn't a widely accepted description of this occurrence [6]. Three general categories can be used to classify traffic congestion: (i) capacity-related, (ii) delay-related, and (iii) cost-related. When traffic flow on a transit system is characterized by higher densities and slower speeds in comparison to a baseline state that has been chosen and has low densities and high speeds, it is said to be congested due to capacity. When too many cars are using a particular stretch of road at one time, the result is congestion, which slows down traffic considerably and creates “free flow” or normal speed delays. The extra expenses brought on by interactions and disturbances among roadway network users are referred to as “cost-associated traffic congestion”. In [14] delineated three fundamental factors that are necessary to evaluate congestion. Among these are the requirements that measure (i) cover the full range of highway performance, (ii) make use of easily available data, and (iii) allow for comparisons across various urban areas.

The four primary kinds of traffic congestion measurements are: (i) fundamental metrics; (ii) ratio metrics; (iii) level of service; and (iv) indices. The pros and disadvantages of several criteria for evaluating congestion have been listed and assessed. In our experiment, we used the above kinds of metrics for calculating the traffic congestion in the given city.

### 4.1 Fundamental metrics

Delays are estimated using fundamental metrics. When a road user must spend more time than they would if they were traveling freely or for a fair period, this is referred to as a delay. Three metrics of segment delay, as determined by Equations (3) and (4), congested travel, which takes into account the length of a crowded street, were weighted by quantity or individual segment delays. All of these measures work together to evaluate individual segment latency. The total delay within a corridor or urban area, whether it relates to volume or person-weighted traffic delay, is then computed by adding up the delays for each specific segment.

(3) $$D_{s}=[TT_{ac}-TT_{ap}]\times V_{p}$$

(4) $$D_{s}^{\prime }=[TT_{ac}-TT_{ap}]\times V_{p}\times V_{oc}$$

In the above equations, $D_{s}=$ segment delay in mins for vehicles; $D_{s}^{\prime }=$ segment delay in mins for persons; $TT_{ac}=$ mins of real journey; $TT_{ap}=$ allowable time for journey (in mins); $V_{p}=$ volume of automobiles during the most hectic time; $V_{oc}=$ capacity of vehicles (people/vehicles).

### 4.2 Ratio metrics

One trip time or delay element is typically divided by another to create ratio estimates of congestion in traffic. Based on travel rate, created several ratio metrics, comprised of the following: ratio, relative, and actual delay rates. The speed at which a road segment is traveled was determined to be the travel rate (measured in mins per mile). The highest rate of travel (or the slowest pace) at which a segment can be covered or a journey can be finished without causing an intolerable degree of motion was referred to as the acceptable travel rate. Using the following Equations (5), (6), and (7).

(5) $$DR=TR_{ac}-TR_{ap}$$

(6) $$RDR=\frac{DR}{TR_{ap}}$$

(7) $$DRA=\frac{DR}{TR_{ac}}$$

In the above equations, travel rate, $TR=TT/L_{s}=60/V$; TT= mins spent travelling; $L_{s}=$ length of section (in miles); *V*= velocity of journey (mph); $TR_{ac}=$ real pace of travel (mins / mile); $TR_{ap}=$ appropriate pace of travel (mins / mile).

### 4.3 Level of service (LOS)

In the theory and execution of transport strategies today, along with the development of numerous forms of transport infrastructure, the LOS has evolved into a standard for evaluating the operating quality (HCM, 2010; TCQSM, 1999) [16]. Over the past 20 years, various iterations of Highway Capacity Manuals, including HCM 2000 and HCM 2010, have been created for every aspect of the road network, including pedestrian and bicycle connections, public passenger transportation, as well as all kinds of junctions, two-lane road portions, city street parts, and motorway segments. The following metrics, as per the IATA concept [7], are used to evaluate the LOS provided to passengers at airport terminals:

• Ideal amount of space for each person.
• Ideal duration for waiting.

The examination of contemporary rules for transport planning and designing infrastructure permits the contention that criteria like service quality have become essential to them. The LOS indicator is widely used, therefore, it makes sense to apply it to UPT intermodal hubs as well. This would add the hubs to the list of urban transport infrastructure elements that are given a consistent evaluation technique. As a result, the total time spent on transfers T(S) is used to objectively measure the LOS:

(8) $$T=t_{p}+t_{w}$$

In the above Equation (8), $t_{p}$ is the travel time between the embarking and departure points; $t_{w}$ is the waiting time for a transfer.

### 4.4 Indices

Several factors connected to traffic congestion have been combined into an equation by certain researchers to provide index assessments of the condition. In [29] created a congestion index as a gauge of overcrowding. The human movement speed value was divided by a standard value to get the corridor mobility index. The speed of person movement, which is commonly stated in person miles per hour, was calculated by multiplying the number of passengers transported by a typical speed on a specific path. Even though the majority of the indices have broad applicability across urban areas, they have the following shortcomings [15]:

• The congestion index can only be applied to a specific route or portion of a roadway.
• The whole metropolitan region cannot be covered by the corridor mobility index; its usage is restricted to a specific corridor.

**Figure 6:**
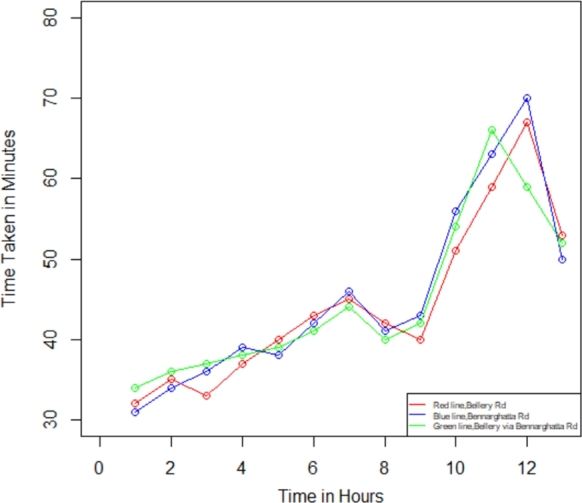
Traffic in weekdays on initial period.

**Figure 7:**
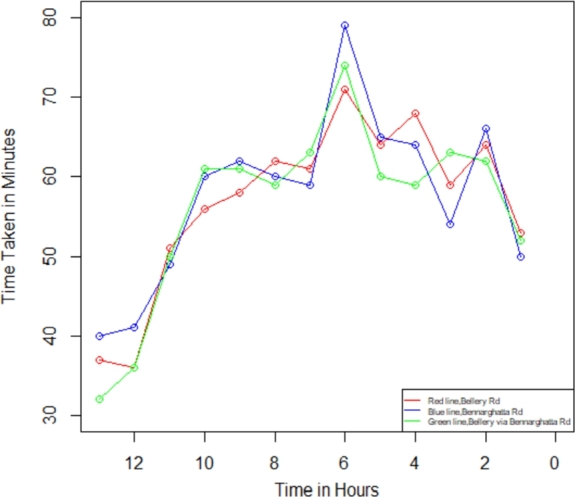
Traffic in weekdays on final period.

**Figure 8:**
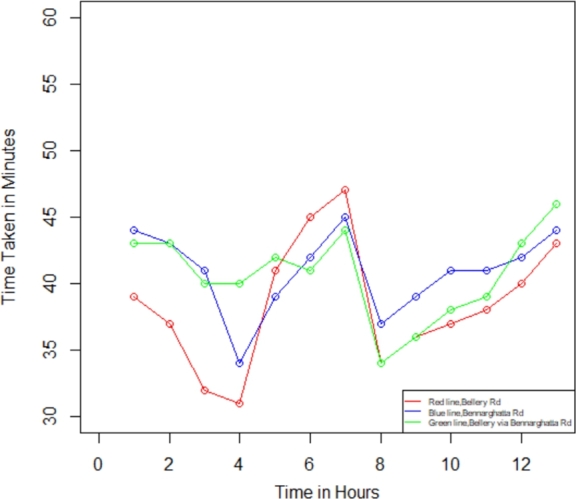
Traffic in weekends on Initial Period.

**Figure 9:**
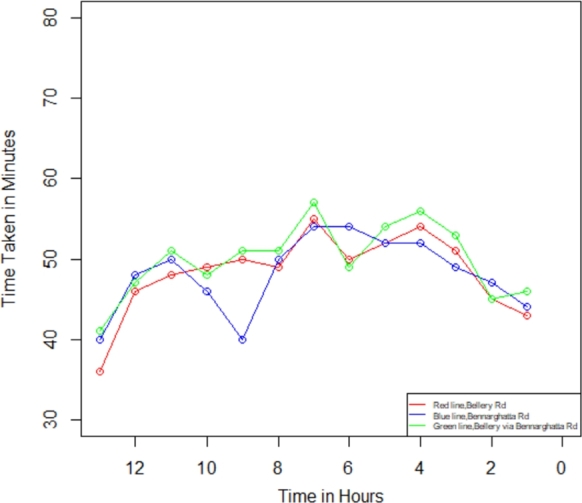
Traffic in weekends on final period.

In the present investigation, we compute the degree of traffic jams in Bengaluru city between the two specified places using Equations (5) - (7). We utilize the R Studio program to determine the traffic congestion level after the computation, followed by employing the outcome values to figure out the plot-wise shown in Figure 6, Figure 7, Figure 8, Figure 9. We have assessed the measurements in two categories within this investigation: weekdays and weekends. Additionally, we have segmented these into two intervals: the initial period spans from 12:00 a.m. to 12:00 p.m., while the final period spans from 12:00 p.m. to 12:00 a.m., respectively, to identify the traffic congestion for three different routes.

## 5 Metric for fuel prices

The cost of fuel, parking, road tolls, and public transportation are all considered part of the cost of transportation. In [15] the proportion of fuel expenses to the total cost of driving, and gasoline prices are very important. To manage traffic flows, ease traffic bottlenecks, increase road safety, and lower atmospheric pollution, it is essential to comprehend how drivers react to changes in gasoline prices. During off-peak hours on weekdays and weekends, there is a negative correlation between the petrol price and traffic flows. The results show that there is a positive elasticity during weekday peak hours, indicating that increased petrol costs may mitigate hyper congestion during these periods. Fuel costs have a positive correlation with public transportation use, and the proportional impact of changes in petrol prices on the quantity of fuel demanded is greater than the impact on the flow of traffic. We use the following method to investigate the fuel expenses between the two terminals in this study.

### 5.1 Fuel cost

Fuel cost is determined by taking into account both the price and usage of fuel and evaluating the total cost for a specific volume of gasoline. It is the financial analysis of the money spent on fuel to run an equipment, system, or car over a given distance or period. Utilize the equation provided beneath it.

(9) Fuel Cost=(Distance / Fuel Efficiency)⁎Fuel Price

In the equation above, Distance refers to the measurement of the space or gap between two given points; Fuel efficiency refers to the effectiveness with which a vehicle or system uses fuel, commonly indicated by metrics such as miles per gallon (MPG) or similar measures; Fuel price represents the expenditure per unit (like per gallon or liter) for a particular fuel type, such as gasoline or diesel.

This computation just provides an estimate; actual expenses could differ depending on the type of driving circumstances, the exact route chosen, and changes in fuel prices.

### 5.2 Fuel required

The process of calculating fuel required entails figuring out the quantity of fuel needed to travel a designated distance or accomplish a specific task using a vehicle or machinery. This computation relies on the fuel efficiency of the vehicle, denoted as the distance it can travel per unit of fuel, such as kilometers per liter or miles per gallon. The formula used to calculate the fuel required is as follows:

(10) $$\text{Fuel Required}=\frac{\text{Distance Traveled}}{\text{Fuel Efficiency}}$$

In the formula above, Fuel required denotes the quantity of fuel necessary; Distance traveled refers to the distance that needs to be traversed or covered; Fuel efficiency refers to the effectiveness of a vehicle or machinery in terms of the distance it can cover for a given amount of fuel.

This computation assists in strategizing fuel usage and predicting the resources needed for a specific journey or task, promoting effective and economical utilization of fuel resources.

## 6 Results and discussion

There are roughly 5x1010 different routes with Hamilton circuit criterion, according to the graph model shown in Fig. 3. However in this study, route searching was limited to an urban transport direction that returned to the site of origin. That is, the present route is still used by city transport to get from the $1^{st}$ point (Terminal JP Nagar) to the $34^{th}$ point (Hebbal), hence route searching is limited to the return trip from the $34^{th}$ point to the $1^{st}$ point. The overall number of alternate routes is therefore far lower than it was before.

**Figure 10:**
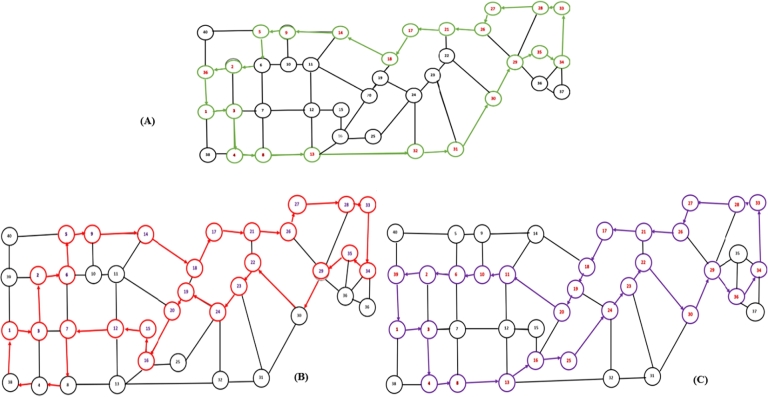
Three alternative optimal routes adhering to the Hamilton circuit criteria on the city transportation route from ‘JP Nagar to Hebbal’ in Bengaluru.

**Figure 11:**
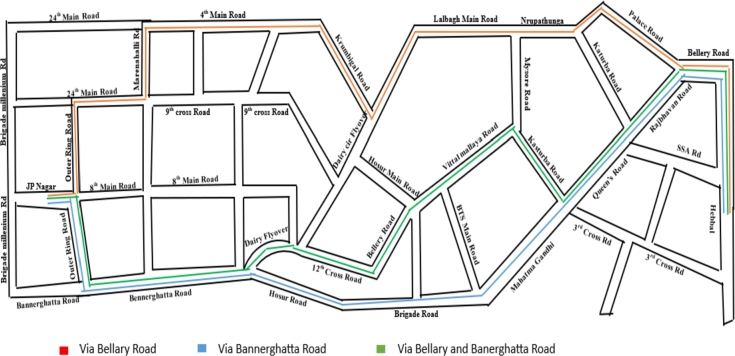
In the actual city strategies, there are three optimal approaches.

It is evident from Fig. 10-((A)-(C)) that the city transit system is now circular rather than a straight path. Moreover, this pathway facilitates an equitable dispersion of road infrastructure, leading to a reduction in congestion typically experienced on frequently traveled urban routes by public transportation. Twelve best course options were derived from the simulation data utilizing the Backtracking method and Hamilton circuit methodology. However, this paper does not detail the twelve routes mentioned; instead, we have identified the top three routes as shown in Fig. 11. These optimal routes are recommended by the Moovit app, a transportation and route-finding application. Subsequently, we analyzed various parameters to determine the most favorable routes (see Table 3).

**Table 3:** Comparison of three routes using different parameters.

Parameters												
Week Days							Week Ends					
	Initial Period			Final Period			Initial Period			**Final Period**		
	Route 1	Route 2	Route 3	Route1	Route 2	Route 3	Route 1	Route 2	Route 3	Route 1	Route 2	Route 3
Distance	Low	Medium	High	Low	Medium	High	Low	Medium	High	Low	Medium	High
Time	Low	High	Medium	Medium	High	Low	Low	High	Medium	Medium	Low	High
Traffic level	Medium	High	Low	Low	High	Medium	High	Medium	Low	Medium	Low	High
Fuel required	Low	Medium	High	Low	Medium	High	Low	Medium	High	Low	Medium	High
Spare	Low	Medium	High	Low	Medium	High	Low	Medium	High	Low	Medium	High

In this research, we explore the diverse routes from JP Nagar to Hebbal, considering different pathways. The distances vary based on the roads taken. Specifically, the distance via Bellary Road is 17.7 km, via Bannerghatta Road is 19.5 km, and via both Bellary Road and Bannerghatta Road is 19.7 km and those reached via routes 1, 2, and 3. These distances are categorized as low, medium, and high. The public transportation covers a distance of 7 kilometers per liter of fuel. Subsequently, we analyze the three routes, dividing them into two segments for weekdays and weekends, and further categorize them into initial and final periods. We examined the fuel needed for the specified distances, computed through the application of Equation (9), (10). Additionally, the amount of petrol needed for travel along the specified routes from JP Nagar to Hebbal is 2.53, 2.79, and 2.81 liters, respectively.

Following the analysis, it has been determined that the most optimal route from JP Nagar to Hebbal, which goes via Bellary Road, is deemed superior for all calculations. Therefore, we have selected this route as the benchmark for comparison against others.

## 7 Conclusion and future work

The design of traffic modeling techniques can make use of the Hamilton circuit model and the Backtracking approach to determine the best route search for city-based public transport, taking into account trial results and plan information. It is feasible to lessen the degree of saturation on some road portions, which lowers the amount of roadway weight when there is a circular alternate route available. Twelve other possible paths along Bengaluru's “JP Nagar - Hebbal” urban transit route constitute the findings from the study, and each of these routes satisfies the requirements to be considered a Hamilton circuit. Hence, in summary, we have assessed different factors and identified the optimal route, designated as Route 1, for daily commuting within Bengaluru city. This process presents a significant challenge due to the ever-growing transportation complexities, especially in Bengaluru, known as one of the most congested urban centers in India. We also present the list of problems as future work.

1. Exploring optimal route between two important destinations in other major cities in India and other countries using the Hamilton circuit algorithm.

2. Optimizing Bengaluru's transport routes using advanced ML and DL methods such as SVR, Random forest, Gradient boosting, RNNs, CNNs, and GNN to enhance route forecasting and dynamic optimization.

By implementing these ML and DL approaches, Bengaluru may significantly improve its public transport routes, making urban transportation more efficient, adaptive, and user-friendly.

## Data and code availability statement

Data and code will be made on reasonable request to the corresponding author.

## Ethics statement

Not applicable for the current study.

## CRediT authorship contribution statement

**Parkavi S:** Writing – original draft. **Parthiban A:** Writing – review & editing, Supervision.

## Declaration of Competing Interest

The authors declare that they have no known competing financial interests or personal relationships that could have appeared to influence the work reported in this paper.
